# Cirrhosis With Splenomegaly and Pancytopenia Complicating a Concurrent Diagnosis of Acute Lymphoblastic Leukemia

**DOI:** 10.7759/cureus.31707

**Published:** 2022-11-20

**Authors:** Swathi Kanakamedala, Shivang U Danak, Earl J Conway, Venumadhav Kotla

**Affiliations:** 1 Department of Internal Medicine, Northeast Georgia Medical Center Gainesville, Gainesville, USA; 2 Department of Medicine, Division of Hematology & Oncology, University of Florida, Gainesville, USA; 3 Department of Pathology, Northeast Georgia Medical Center Gainesville, Gainesville, USA; 4 Department of Hematology and Oncology, Longstreet Clinic Cancer Center, Gainesville, USA

**Keywords:** bone marrow, cirrhosis, splenomegaly, acute lymphoblastic leukemia, pancytopenia

## Abstract

Pancytopenia, a hematologic condition, is a decrease in all three blood cell lines. The two main etiologies include decreased production or increased destruction of cells, as seen in nutritional deficiencies or liver cirrhosis, respectively. Pancytopenia commonly presents with fever, splenomegaly, and lymphadenopathy. Initial workup includes complete blood count, metabolic panel, peripheral smear, anemia panel, erythrocyte sedimentation rate, C-reactive protein, and lactate dehydrogenase. Workup also involves excluding toxins, human immunodeficiency virus (HIV), drug effects, and infectious etiologies. Malignancies can cause impaired production of cell lines. For hematologic malignancies, a bone marrow biopsy is performed. In patients above the age of 55 who are diagnosed with acute leukemia, acute lymphoblastic leukemia (ALL) is known to make up approximately 20% of all cases. Furthermore, ALL requires the presence of more than 20% lymphoblasts seen on bone marrow biopsy. Treatment includes induction, consolidation, and maintenance chemotherapy.

We report the case of a 63-year-old male with a history of liver cirrhosis from non-alcoholic fatty liver disease who presented for consultation due to pancytopenia without signs of fever or lymphadenopathy. Imaging revealed cirrhosis, ascites, and moderate splenomegaly while the workup for toxins, infections, and HIV was negative. He presented to the hospital with worsening anasarca and acutely worsening pancytopenia. Peripheral smear showed pancytopenia with no definitive blasts, whereas bone marrow biopsy revealed B-lymphoblastic leukemia. He was transferred to a tertiary center for induction chemotherapy but ultimately transitioned to supportive care due to intolerance.

This case demonstrates the importance of having a high suspicion for leukemia with an acute decline in all three cell lines, thereby prompting a bone marrow biopsy. Although lacking in the literature, adult patients with ALL can present with splenomegaly without fever or lymphadenopathy. These examination findings are clinical clues to evaluate for underlying malignancies in patients with pancytopenia, although coexisting etiologies may exist. Lastly, peripheral smear alone is insufficient to screen for diagnosis of ALL as it can be normal despite bone marrow involvement.

## Introduction

Pancytopenia is a hematologic condition defined as a decrease in all three blood cell lines and is characterized as hemoglobin (Hb) less than 12 g/dL in women and 13 g/dL in men, leukocytes less than 4,000/mL, and platelets less than 150,000/µL [1]. Pancytopenia itself is not a disease but rather a manifestation of other conditions. Two main etiologies of pancytopenia include conditions that cause decreased production of cells or increased destruction of cells. A decreased production of cells is often secondary to nutritional deficiencies, whereas an increased destruction of cells is often secondary to various autoimmune conditions, alcoholic liver cirrhosis, and human immunodeficiency virus (HIV) [1]. See Appendix 1 for etiologies of pancytopenia.

Pancytopenia can also be seen due to underlying liver cirrhosis. The pathogenesis is multifactorial, including portal hypertension, splenic sequestration, alterations in thrombopoietin, and bone marrow suppression mediated by toxins [2]. Chronic liver cirrhosis is associated with multiple complications, such as splenomegaly and hypersplenism. Hypersplenism itself is thought to be a cause of cytopenia and thrombocytopenia in liver cirrhosis [3]. Liver cirrhosis leads to portal hypertension which causes increased congestion in the portal system, leading to splenomegaly. In addition, the spleen influences liver cirrhosis progression by modulating hepatic fibrogenesis, immune microenvironment dysregulation, and liver regeneration [3].

The first step in the investigation of pancytopenia often includes ruling out the effects of drugs or toxins. Pertinent lab work includes complete blood count (CBC), comprehensive metabolic panel, peripheral smear, iron studies, reticulocyte count, erythrocyte sedimentation rate (ESR), C-reactive protein (CRP), and lactate dehydrogenase (LDH) [4]. Infectious workup includes HIV, hepatitis panel, and viral or fungal causes [5]. Additional workups including copper, zinc, Coombs test, vitamin B12, reticulated platelets, haptoglobin, folate levels, and antinuclear antibodies (ANA) can help differentiate consumption disorders versus production disorders versus peripheral destruction and impaired production [4]. Refer to Appendix 2 for lab work for pancytopenia. Bone marrow biopsy is indicated if there is no clear etiology or if the suspicion of non-nutritional production disorders is high [4]. Additionally, if there is a concern for hematologic malignancy then flow cytometry and cytogenetics can be obtained on the bone marrow aspirate [5].

Malignancies, such as lymphomas and leukemias, can cause impaired production of cell lines which can also result in pancytopenia. Metastatic tumors can cause bone marrow replacement which will eventually result in pancytopenia [1].

Acute lymphoblastic leukemia (ALL) is most common in children and accounts for 75% of all acute leukemias in children [6]. The percentage is much lower in adults, with only 20% of patients diagnosed after the age of 55 years [7]. In contrast to children, ALL in adults is associated with higher mortality. The clinical onset of ALL is often acute and presents with symptoms of fever, fatigue, lethargy, bone pain, and bleeding [7]. ALL presents with two types of lymphoid lineage, namely, T-cell ALL and B-cell ALL. Patients who have T-cell ALL often present with respiratory distress secondary to mediastinal mass that may or may not be associated with pleural effusions [8]. Conversely, large abdominal lymph nodes are found in the B-cell subtype of ALL, which generally accounts for only 5% of B-cell ALL [5]. Classification of ALL is based on immunophenotypic and genetic analysis [9].

The diagnostic approach includes a comprehensive history and physical examination that is focused on identifying the severity of symptoms and the presence of risk factors. Risk factors that can have an impact on therapeutic decisions include prior exposure to chemotherapy, HIV, hepatitis viruses, and coexisting medical conditions [8]. Initial workup includes CBC with peripheral smear and comprehensive metabolic panel, as well as coagulation studies [8]. According to the National Comprehensive Cancer Network (NCCN) guidelines, ALL requires the presence of at least 20% or more lymphoblasts identified on bone marrow aspirate and biopsy, flow cytometric immunophenotyping, minimal residual disease analysis, and karyotyping. Further risk stratification is recommended by the NCCN through cytogenetics and fluorescence in situ hybridization (FISH).

Treatment for ALL involves induction chemotherapy with a four-drug regimen (vincristine, prednisone, an anthracycline, cyclophosphamide or L-asparaginase) or a five-drug regimen (vincristine, prednisone, an anthracycline, cyclophosphamide) plus asparaginase over four to six weeks. Complete remission is obtained in 65-85% of patients. If remission is obtained, the next phase is consolidation chemotherapy or stem cell transplant. After consolidation, patients can be treated with maintenance chemotherapy [10].

## Case presentation

A 63-year-old male with a medical history of arthritis, basal cell carcinoma, non-diabetic, and liver cirrhosis due to non-alcoholic fatty liver disease was referred to hematology and oncology due to findings of pancytopenia. He denied any history of alcohol or tobacco abuse. He reported a history of receiving blood transfusions. His only medications were vitamin B12 and magnesium supplements. He denied bleeding, epistaxis, weight loss, or any febrile illnesses.

Labs prior to the visit showed iron 239, total iron-binding capacity (TIBC) 249, iron saturation percentage 96, ferritin 350, reticulocyte count 2.2, white blood cell (WBC) 2.7, absolute neutrophil count (ANC) 1,570, Hb 11.6 g/dL, mean corpuscular volume (MCV) 97.9, platelets 39,000, and his hepatitis panel was negative. 

During the initial clinic visit, his medical history and labs were reviewed. Given elevated ferritin and a history of fatty liver, a hereditary hemochromatosis test was ordered along with a repeat CBC. An abdominal ultrasound was ordered to evaluate for cirrhosis and hypersplenism. If pancytopenia was not attributable to liver cirrhosis, the idea of pursuing a bone marrow biopsy to rule out primary hematologic conditions was also discussed with the patient. He was instructed to return to the clinic in one month.

The abdominal ultrasound showed cirrhotic changes with a small nodular liver, a mild amount of ascites, and moderate splenomegaly, measuring 21 × 14 × 6 cm.

However, approximately two and a half weeks later, the patient presented to the emergency department with lower extremity swelling and pain.

He reported the swelling had started four days prior, with pain nine out of ten, worse with walking, and alleviated by lying down. Lab work showed significant pancytopenia with WBC 2.2 (ANC 1,170), Hb 7.7 g/dL, and platelets 51,000. Total bilirubin was 8.5, and the international normalized ratio (INR) was 1.74. The patient was subsequently admitted to the hospital. A venous duplex scan (VDS) of the lower extremity revealed a chronic deep vein thrombosis (DVT). Given thrombocytopenia, anticoagulation was not started. Gastroenterology was consulted in setting hyperbilirubinemia and pancytopenia. Magnetic resonance imaging (MRI) of the abdomen showed hepatic cirrhosis, splenomegaly (measuring 18.2 cm in craniocaudal dimension), gastric and esophageal varices, and ascites. He was subsequently started on furosemide and spironolactone.

Given indirect hyperbilirubinemia and pancytopenia, additional workup revealed a negative Coombs test and HIV screen, haptoglobin 10, LDH 416, ESR 23, and CRP 10.30. Anemia profile showed ferritin 475, iron 183, iron saturation 82, TIBC 223, folate 9.73, and vitamin B12 820. Over the next 24 to 48 hours, despite being started on empiric antibiotics for cellulitis, his pancytopenia notably worsened. He developed neutropenic fever, with an ANC of 300, and Infectious Disease was consulted. Blood cultures from admission showed no growth and antibiotics were changed to ceftriaxone. Peripheral smear showed pancytopenia with no definitive blasts. Thereafter, a bone marrow biopsy was performed and revealed B-cell ALL.

**Figure 1:**
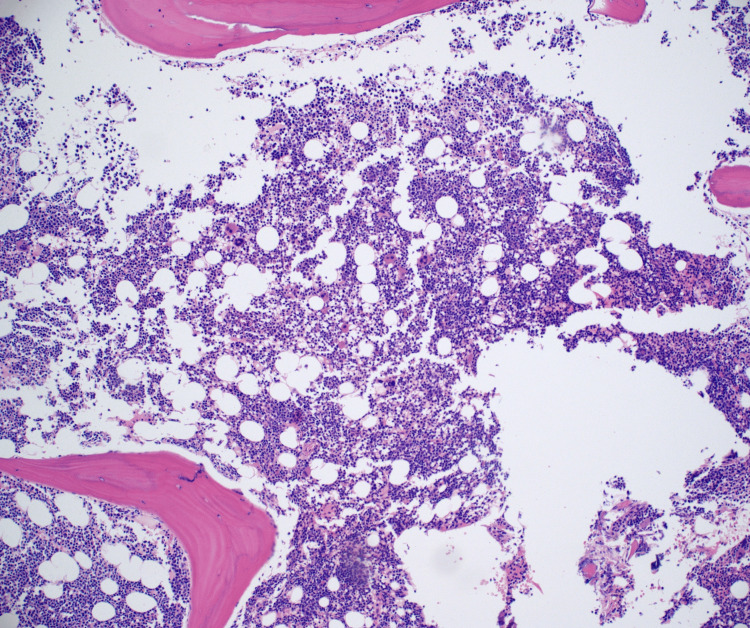
Hematoxylin and eosin (100×): hypercellular bone marrow for age approximately 70% with infiltrate.

**Figure 2:**
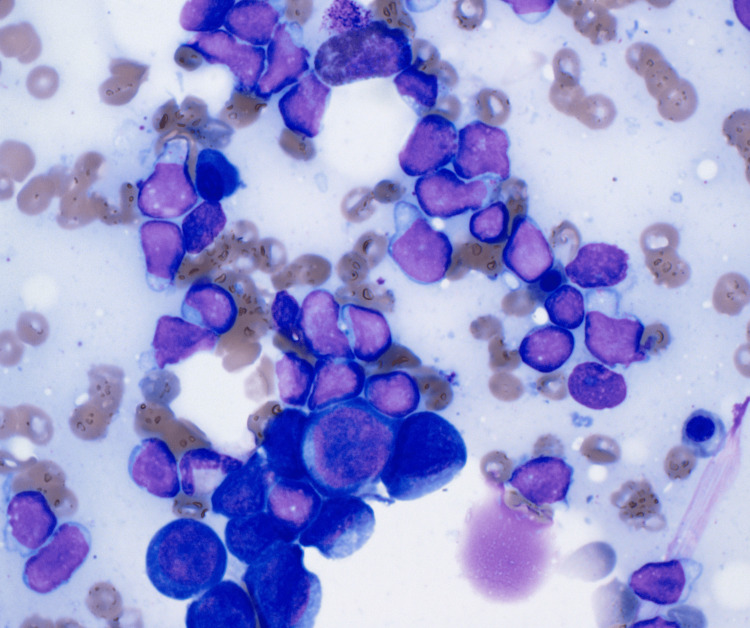
Wright Giemsa (1000×): B-lymphoblasts and erythroid hyperplasia.

**Figure 3:**
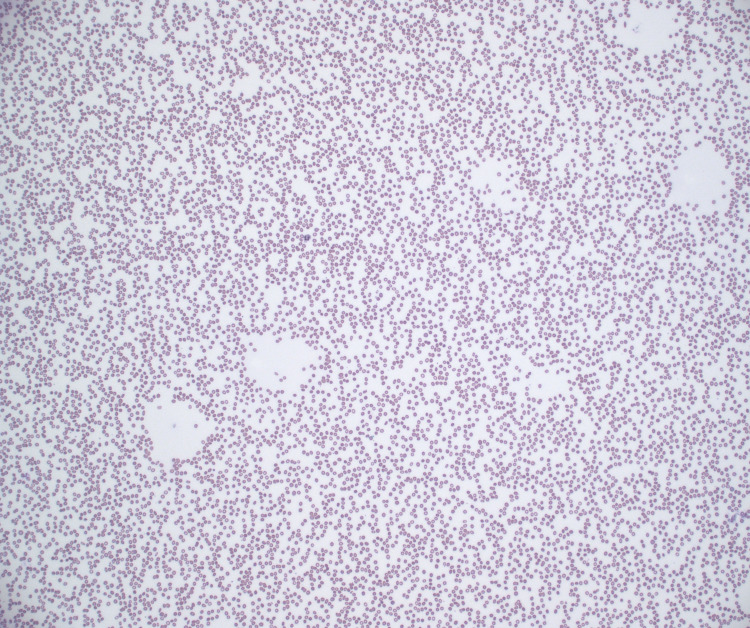
Wright Giemsa (100×): severe pancytopenia with only a single white blood cell noted in the field.

**Figure 4:**
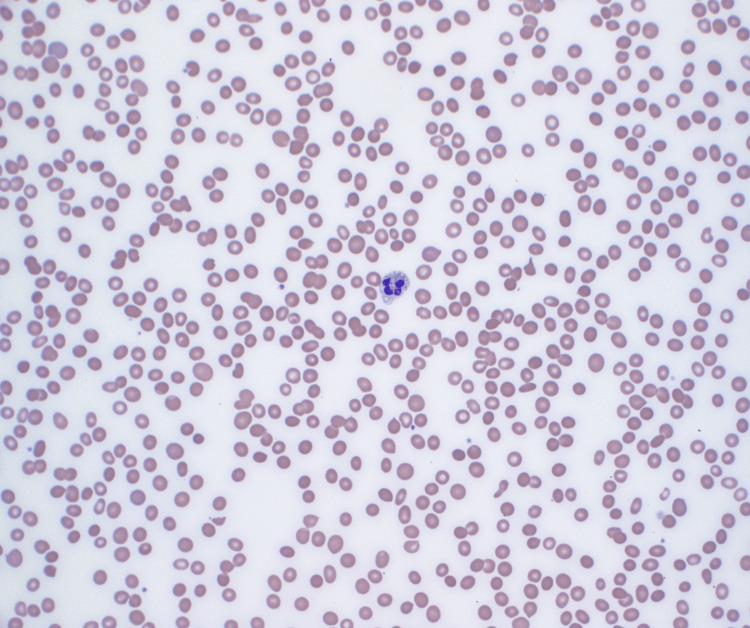
Wright Giemsa (400×): severe pancytopenia with only a single white blood cell noted in the field.

Hematoxylin and eosin stain showed hypercellular bone marrow with 70% infiltrate (Figure 1). Wright Giemsa stain showed B-lymphoblasts, erythroid hyperplasia, and severe pancytopenia (Figures 2-4).

Flow cytometry showed a prominent blast population constituting 81% of total analyzed cells, which expressed CD10, CD13, CD19, CD22, CD33, CD38 (variable, dim to moderate), CD45, HLA-DR (variable, dim to moderate), Tdt, and cytoplasmic CD79a.

**Figure 5:**
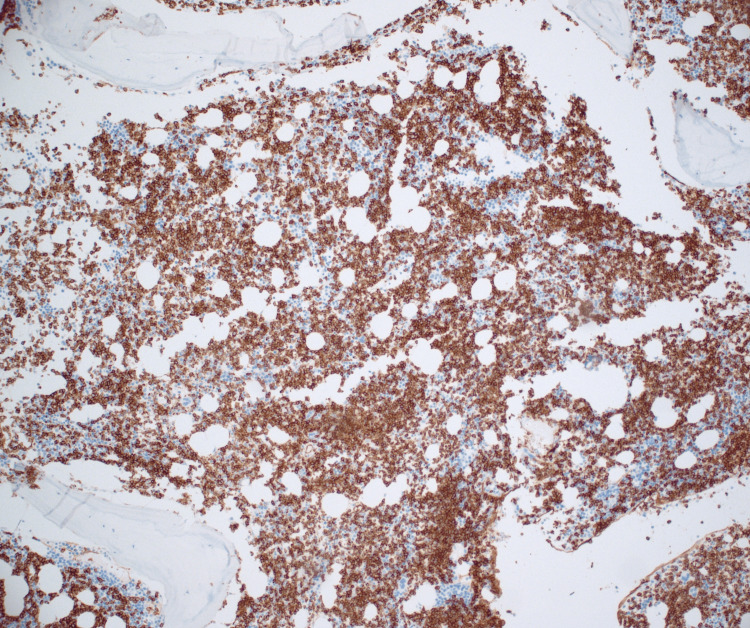
CD34 Immunohistochemical analysis (100×): hypercellular bone marrow for age approximately 70% with infiltrate.

**Figure 6:**
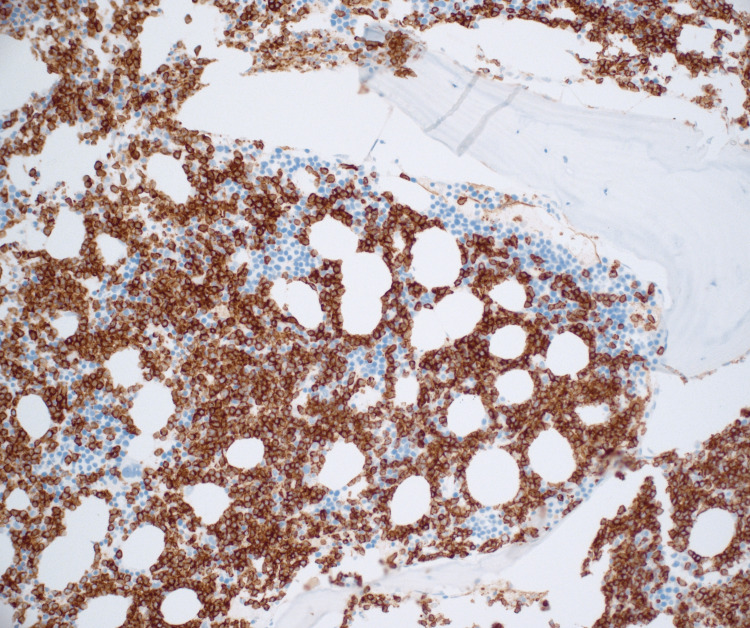
CD34 Immunohistochemical analysis (400×): hypercellular bone marrow for age approximately 70% with infiltrate.

Immunohistochemistry revealed staining for CD34 showing that the majority of the cells present (greater than 80%) were CD34-positive blasts and co-expressed B-cell marker PAX-5 (Figures 5, 6). The cells were CD3 negative. Staining for CD71 and CD15 showed decreased background hematopoietic elements with markedly decreased myeloid and inverted M:E ratio of less than 0.5:1.

Cytogenetics and FISH analysis from the bone marrow biopsy was positive for CRLF2 rearrangement (partial deletion), and negative for Trisomy 4, 10, and 17; for MYC, KMT2A, and IGH rearrangements; for BCR::ABL1 (9;22) and ETV6::RUNX1 (12;21) fusions; and for deletion of 9p21. These findings were compatible with B-lymphoblastic leukemia/lymphoma (B-ALL).

Given these findings and confirmation of the diagnosis of B-cell ALL, the patient was transferred to a tertiary center for further management and care.

Our case describes a patient whose liver cirrhosis was initially attributed as the etiology of pancytopenia. Nonetheless, during the initial visit with the hematologist, a bone marrow evaluation was suggested to the patient, but not pursued at that time. The acute worsening of pancytopenia during his hospitalization suggested that there may be another underlying etiology contributing to the depressed cell lines. As a result, a bone marrow biopsy along with additional lab work was performed.

The peripheral blood smear showed pancytopenia and no definitive blasts were identified. Bone marrow biopsy finally revealed B-lymphoblastic leukemia/lymphoma, thereby revealing the underlying pathogenesis and etiology of the pancytopenia.

## Discussion

The most common presenting clinical features of ALL are febrile illness and lymphadenopathy, but splenomegaly can be an underlying feature as well, although less common in adults compared to children. Sultan et al. (2016) performed a cross-sectional study of 51 cases of adult ALL demonstrating that lymphadenopathy was the predominant finding in 43.1%, followed by splenomegaly (23.5%) and hepatomegaly (21.5%) [11].

Our patient’s initial presentation appeared more consistent with underlying liver disease. He did not have lymphadenopathy but did have splenomegaly. Without a high index of suspicion for an underlying marrow disorder, it would not be necessary to obtain a bone marrow biopsy immediately. The abrupt and persistent decrease in all three cell lines supported the likelihood that the pancytopenia was likely due to an underproduction etiology, rather than a consumption etiology. Although liver cirrhosis causing pancytopenia is multifactorial and tends to lean toward a decreased production issue, it is uncommon for all three cell lines to drop abruptly. Upon literature review, there is limited data to demonstrate an association between cirrhosis and leukemia as presenting with pancytopenia.

Additionally, our patient had moderate splenomegaly identified on imaging, which was attributed to his cirrhosis. Although splenomegaly is present in 69% of children with ALL at diagnosis, splenomegaly is only present in 48% of adults with ALL [12]. Friedman et al. (1981) reported splenomegaly to be an adverse prognostic factor [12].

Although there may be similar cases of splenomegaly and adult ALL, the current literature lacks case reports on this topic. Our case highlights the importance of maintaining a high index of suspicion of underlying leukemia despite other attributable causes of pancytopenia. Most notably, physical examination findings such as splenomegaly and lymphadenopathy may also provide clues to an alternative diagnosis.

Ultimately, the underlying diagnosis of cirrhosis complicated treatment decisions and the patient did not tolerate the standard intensive multi-agent chemotherapy. Within a brief period, he transitioned to supportive care only.

## Conclusions

In summary, a peripheral smear review alone is insufficient to screen for a diagnosis of ALL as it can be normal despite bone marrow involvement. With the understanding of the indolent and slow progression of cytopenias in the setting of hypersplenism from cirrhosis, a rapid decline in all three counts over a brief period should prompt clinical investigation for alternate etiologies such as medications, infections, and neoplastic conditions. If those are unrevealing, a rapid decline in all three cell lines should prompt a bone marrow biopsy in the investigation of pancytopenia.

## Appendices

Appendix 1

**Table 1:** Causes of pancytopenia based on bone marrow infiltration, bone marrow failure, and destruction/sequestration. SLE: systemic lupus erythematosus; RA: rheumatoid arthritis; HIV: human immunodeficiency virus; EBV: Epstein-Barr virus; DIC: disseminated intravascular coagulation

Causes of pancytopenia	
Bone marrow infiltration/replacement	Acute leukemia
	Chronic leukemia
	Myelodysplastic syndromes
	Metastatic disease
	Infectious
	Myelofibrosis
Bone marrow failure	Aplastic anemia
	Medications – cytotoxic drugs
	Autoimmune disorders (SLE, RA, sarcoidosis)
	Megaloblastic (vitamin B12, folate)
	Excessive alcohol
	Deficiencies – copper, zinc
	Malnutrition
	Viral infection (HIV, hepatitis, EBV)
Destruction/Sequestration/Redistribution	Consumption – DIC
	Splenomegaly – portal hypertension/cirrhosis, infections, autoimmune disorders, malignancies

Appendix 2

**Table 2:** Relevant lab work for the workup of pancytopenia CBC: complete blood count; WBC: white blood cell; RBC: red blood cell; PT: prothrombin time; PTT: partial thromboplastin time; ESR: erythrocyte sedimentation rate; CRP: C-reactive protein; LDH: lactate dehydrogenase

Workup of pancytopenia	
Lab work	CBC – WBC and RBC
	Peripheral blood smear
	Reticulocyte count (low value supports hypoproliferation)
	PT and PTT
	Serum chemistry (electrolytes, renal and liver function tests)
	ESR, CRP, LDH, calcium, uric acid
	Blood type and screen
